# Embodied Speech: Sensorimotor Contributions to Native and Non-Native Phoneme Processing and Learning

**DOI:** 10.1162/NOL.a.215

**Published:** 2026-01-22

**Authors:** Tzuyi Tseng, Jennifer Krzonowski, Claudio Brozzoli, Alice C. Roy, Véronique Boulenger

**Affiliations:** CNRS, Université Lyon 2, Laboratoire Dynamique du Langage, Lyon, France; Integrative Multisensory Perception Action & Cognition Team (ImpAct), Centre de Recherche en Neurosciences de Lyon, INSERM, Université Claude Bernard 1, Lyon, France

**Keywords:** embodiment, foreign language learning, manual gestures, motor system, non-native phonemes, speech perception

## Abstract

Learning to recognize and produce foreign speech sounds can be challenging, particularly when only subtle differences distinguish these new sounds from phonemes in the native language. Functional neuroimaging evidence shows that the motor cortex is involved in speech production and in perceptual phonemic processing. This highlights the embodied nature of speech perception, predicting the potential benefits of sensorimotor-based training approaches to enhance the acquisition of foreign speech sounds. Hence, here we first review current findings on the motor contribution to not only native but also non-native phoneme perception. Available evidence has established that motor cortical activity especially shows up under non-optimal perceptual conditions, such as when native phonemes are degraded by noise or when listeners perceive non-native speech sounds. Drawing upon this evidence, we then review training paradigms that have been developed for learning foreign phonemes, with a special emphasis on those embedding manual gestures as cues to represent phonetic features of the to-be-learned speech sounds. By pointing to both strengths and caveats of available studies, this review allows to delineate a clear framework and opens perspectives to optimize foreign phoneme learning, and ultimately support perception and production.

## INTRODUCTION

Over the last 15 years, research on language production and comprehension has undergone a conceptual revolution, highlighting how language may rely not only on specific brain areas but also on embodied processes underpinned by sensorimotor brain regions. A large amount of studies has underlined the sensorimotor grounding of various processes at play in language comprehension, particularly in processing action verbs and understanding sentences (see Fischer & Zwaan, 2008, and Franken et al., 2022, for reviews). However, one crucial aspect of language processing—phoneme perception—has received comparably less attention in the context of embodied cognition. Yet, motor involvement seems to play a significant role in decoding sounds within a specific linguistic code. The focus of the current review is to provide an updated overview of research findings in this domain, focusing on both native and foreign (i.e., non-native) phoneme perception. Additionally, it examines whether and how sensorimotor-based training may enhance phonological processing and learning of foreign language speech sounds.

## MOTOR RESONANCE TO NATIVE SPEECH PERCEPTION

**Figure 1.**
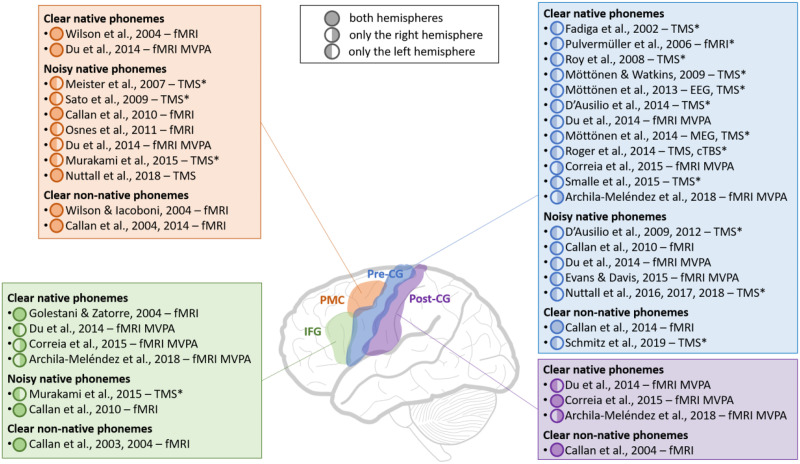
Overview of neuroimaging studies showing the involvement of speech production brain regions in phoneme perception. These regions include the inferior frontal gyrus (IFG, in green), premotor cortex (PMC, in orange), precentral gyrus (pre-CG, in blue), and postcentral gyrus (post-CG, in purple). For each region, the fMRI and TMS studies that investigated phoneme perception under different perceptual conditions (clear native phonemes, noisy native phonemes, and clear non-native phonemes) are indicated in a box of the corresponding color. For each study, a full-colored circle indicates that significant activity was observed in both hemispheres, whereas a half-colored circle indicates significant activity in only one hemisphere. Studies that investigated cortical activity in the left hemisphere only are marked with *. fMRI = functional magnetic resonance imaging; MVPA = multivoxel pattern analysis; TMS = transcranial magnetic stimulation; EEG = electroencephalography; MEG = magnetoencephalography; cTBS = continuous theta-burst stimulation.

Neuroimaging studies using transcranial magnetic stimulation (TMS) or functional magnetic resonance imaging (fMRI) have provided compelling evidence that (pre)motor regions involved in speech production are also activated during the mere perception of native phonemes (see Figure 1 for an overview of brain imaging studies reporting (pre)motor activity during phoneme perception). Brain activity in the motor system echoes the motor theory of speech perception (Liberman et al., 1967; Liberman & Mattingly, 1985; Liberman & Whalen, 2000), suggesting that speech is perceived by decoding the invariant articulatory properties of speech sounds, namely the vocal tract gestures of the speaker used to produce those sounds. According to this view, motor regions responsible for generating speech movements are also engaged during speech perception. In their seminal work testing this theory, Fadiga and colleagues (2002) applied TMS to the left motor cortex of Italian adults while they listened to words and pseudowords featuring either the double lingua-palatal fricative (alveolar trill) /rr/, which requires strong tongue tip movements to be produced, or the double labiodental fricative /ff/, which involves only minimal tongue movements. Corroborating Liberman’s view, items embedded with /rr/ elicited higher tongue motor evoked potentials (MEPs) than the other conditions, showing automatic and somatotopic activity in the motor cortex for passive speech perception, as if articulatory movements were decoded (see also Watkins et al., 2003, for lip-MEPs during listening to or viewing speech). Roy and colleagues (2008) not only replicated this phonological effect with Italian pseudowords including the tongue-related /ll/, but also revealed an early lexical effect, further supporting the link between speech perception and motor activation. When TMS was applied 200 and 300 ms after the double consonant, the mere perception of /ll/ in Italian rare words evoked larger MEPs than /ll/ in frequent words. This finding suggests that the motor cortex not only participates in phonological encoding during speech perception but also interacts with top-down lexical processes. Note that parallels and shared representations between the perception and production systems have also been reported at the word level, with a similar temporal brain dynamic of phonological and lexicosemantic processes between the two modalities (Fairs et al., 2021; Strijkers & Costa, 2012; see also Strijkers et al., 2017). Additional evidence of speech-induced motor activity comes from D’Ausilio and colleagues (2014), who applied ultrasound tissue Doppler imaging (UTDI) to record whole tongue movement synergies evoked by TMS during speech perception. Passively listening to syllables varying in place of articulation and position in the vowel space (/ti/, /to/, /ki/, and /ko/) elicited kinematic patterns that mirrored those of actual phoneme production, with tongue displacement along the anterior-posterior (for coronal /t/ and velar /k/, respectively) and ventral-dorsal (for high-front /i/ and back /o/ vowels, respectively) planes (see also Tang et al., 2021, for disrupted adaptation during production after the perception of altered vowel formants when receiving rTMS on the tongue motor area).

In line with these findings, studies using fMRI also revealed a partial overlap of motor cortical activation during speech perception and production. Wilson and colleagues (2004) pioneered in showing stronger hemodynamic response in the bilateral ventral premotor cortex, typically activated during speech production, when merely perceiving consonant–vowel (CV) syllables, compared to nonspeech sounds (white noise or bell rings). Pulvermüller and colleagues (2006) further supported the specificity of this motor activity, as consonants requiring different places of articulation for production (/p/ or /t/) activated the precentral gyrus somatotopically (lip or tongue motor area, respectively). Other fMRI studies, however, failed to replicate this somatotopic encoding of phonemes in the (pre)motor cortex during passive speech perception. Arsenault and Buchsbaum (2016), for instance, reported equal activity of the lip and tongue motor representations when listening to labial and dental/alveolar consonants. This was confirmed by additional multivariate pattern analyses (MVPA): a classifier trained to distinguish these same consonants from production-related activity in the (pre)motor cortex (i.e., pre/postcentral gyri and central sulcus) was unable to discriminate them when they were only perceived (see also Cheung et al., 2016, for somatotopy of place of articulation in speech production but not perception). Despite these discrepancies, these findings still suggest that articulatory features of perceived phonemes can be decoded from cortical activity in motor and premotor regions. Using a similar MVPA approach, Correia and colleagues (2015) trained a classifier to discriminate between syllable pairs based on either place or manner of articulation, or voicing. They then tested whether the classifier could predict brain activity from other phonemes varying on the same articulatory properties. For instance, they trained a classifier to discriminate place of articulation between labials and dentals in plosives (/pa/ vs. /ta/) and tested its ability to discriminate this same feature in fricatives (/fa/ vs. /sa/). Results revealed that the classifier discriminated both place and manner of articulation from activity patterns in a network distributed over the bilateral postcentral gyrus and the right anterior insula. Generalization to place of articulation further extended to the bilateral superior temporal cortex and the right precentral and inferior frontal gyri. The temporoparietal junction also decoded these two articulatory features, in the left hemisphere for place and in the right for manner of articulation (see also Archila-Meléndez et al., 2018, for converging results on place of articulation). Overall, these studies therefore support that in addition to temporoparietal and inferior frontal regions, premotor and motor areas code for articulatory features even when phonemes are only auditorily perceived, highlighting the sensorimotor nature of speech perception (Liberman et al., 1967; Liberman & Mattingly, 1985; Liberman & Whalen, 2000; Schwartz et al., 2012).

## FUNCTIONAL CONTRIBUTION OF THE MOTOR CORTEX TO SPEECH PERCEPTION

The role of the motor system as an essential or a subsidiary component of speech perception has, however, been hotly debated. The auditory system alone has been argued to be sufficient for decoding speech sounds through the analysis of spectrotemporal acoustic patterns, that is, by using general auditory mechanisms (see Diehl et al., 2004). The dual stream model (Hickok & Poeppel, 2007) also proposed that early speech processing begins bilaterally in auditory regions, to later split into two pathways. While a ventral stream in the temporal lobe would support speech comprehension (i.e., access to meaning), a dorsal stream would translate acoustic signals into articulatory representations in the premotor and inferior frontal cortices, via the parietotemporal junction, with a predominant role in speech acquisition and production. As to speech perception, it is proposed that the dorsal auditory-motor stream would participate only in sublexical processing such as syllable identification, that is, when listeners are required to specifically attend to phonemic information. As advocated by Hickok and his colleagues, motor resources would otherwise not be engaged in naturalistic listening conditions, thus only modestly contributing to speech perception (Hickok, 2012; Hickok & Poeppel, 2004, 2007; Stokes et al., 2019). This perspective is especially supported by clinical findings showing that patients with speech production deficits due to brain lesions in the motor system or the parietotemporal junction often retain intact speech comprehension (Hickok et al., 2011; Rogalsky et al., 2011). Such evidence has led to the interpretation that the neural substrates for speech production may function independently from those for perception.

Building on empirical neuroimaging findings (see below), other theories and models on the contrary support a functional contribution of the motor system to speech perception, emphasizing the sensorimotor nature of speech sounds rather than their purely auditory or motor character (Pulvermüller & Fadiga, 2010; Schomers & Pulvermüller, 2016; Schwartz et al., 2012). Accordingly, the auditory and motor systems would dynamically interact at all stages of speech perception, from acoustic–phonetic to phonemic processing, giving the motor system a primary role (Liebenthal & Möttönen, 2018). Motor regions would in particular instantiate internal forward predictions of the sensory input, influencing auditory processing and constraining phonemic categorization (Iacoboni, 2008; Rauschecker & Scott, 2009; see Grisoni & Pulvermüller, 2022, for a neural marker of phonological prediction in motor areas).

Evidence for tight reciprocal functional links between speech perception and production first comes from behavioral studies. Using electropalatography in healthy adults, Yuen and coworkers (2010) reported that perceiving incongruent distractor speech sounds during syllable production distorted the ongoing articulatory gestures. For instance, pronouncing /ka/ or /sa/ induced closer contact of the tongue against the alveolar ridge while hearing /ta/ rather than the same congruent syllables. This suggests that phoneme perception automatically activates articulatory movements, thus interfering with actual production. Conversely, changes in the articulatory configuration can impact speech perception in adults (Ito et al., 2009) as well as in infants (Bruderer et al., 2015; Choi et al., 2019). At the age of 6 months, temporarily restraining either the tongue tip or the closure of the lips with teething toys indeed impaired the discrimination of non-native dental and labial speech sounds, respectively. This supports an active contribution of motor processes to speech perception early in the course of language development.

TMS studies further provided positive evidence for a causal motor role in speech perception, as temporarily disrupting brain motor areas alters phonemic processing (see Möttönen & Watkins, 2012, for a review). Möttönen and Watkins (2009) found that the identification and discrimination of computer-generated CV syllables lying on a continuum and differing on their place of articulation (labials vs. dentals, e.g., /ba/–/da/ and /pa/–/ta/) were impaired when the lip representation in the left primary motor cortex was momentarily disrupted by repetitive TMS (rTMS). This impairment in categorical perception was not observed when either rTMS was applied over the left-hand motor representation or the perceived speech sounds did not involve the lips to be produced (e.g., /ka/–/ga/ and /da/–/ga/; see also Rogers et al., 2014). A follow-up study using TMS confirmed the motor contribution to the discrimination of speech sounds by showing that brain activity in the lip motor area reflects speech perception per se (i.e., sensitivity for between-category pairs along the continuum) and not post-perceptual decision making or response selection processes (Smalle et al., 2015). In line with this finding, Möttönen and colleagues (2013) reported an effect of motor cortical disruption by rTMS on brain responses to speech sounds, as recorded with electroencephalography (EEG), even in the absence of any behavioral task and when stimuli were not the focus of attention. More precisely, while participants watched a silent movie, temporarily disrupting their lip (but not hand) area in the left motor cortex reduced the early automatic mismatch negativity responses to phonetic changes (infrequent /ba/ or /ga/ in a sequence of /da/), but not to acoustic (duration) changes in speech and non-speech sounds (piano tones). This suggests that the motor system causally affects auditory speech processing when decoding articulatory features of phonemes. Surprisingly, however, the motor interference was not specific to the lip-related phoneme /b/ (relative to /g/), which seems at odds with previous work showing articulatory specific effects (Fadiga et al., 2002; Möttönen & Watkins, 2009). The authors suggested that such effects may depend on attention and particularly occur when participants need to focus on critical phonetic features that are task-relevant. A combined TMS-MEG (magnetoencephalography) study by the same research team corroborated this interpretation (Möttönen et al., 2014): rTMS over the left lip motor area affected the early left-lateralized brain responses to the labial consonant /b/, but not to velar or dental consonants (/g/ or /d/, respectively), when participants were required to respond to these stimuli. In contrast, when those same speech sounds were ignored, lip motor cortical disruption affected bilateral brain responses to the three consonants similarly and in a later time-window after their onset. This suggests that attention facilitates auditory-motor integration in the left hemisphere for processing specific articulatory features of speech sounds, but that left motor regions still automatically interact with bilateral auditory regions in an unspecific manner, that is, irrespective of place of articulation, at a later phonological stage.

Altogether, these findings highlight the close interactive mechanisms at play between the auditory and motor systems in the decoding of speech sounds (Liebenthal & Möttönen, 2018) and provide support for a causal role of motor regions in speech perception.

## THE MOTOR SYSTEM’S FUNCTIONAL ROLE IN CHALLENGING SPEECH PERCEPTION

What is particularly striking from some of the studies reviewed so far is that motor regions seem to be preferentially engaged when speech perception is challenging (Figure 1). D’Ausilio and colleagues (2009) revealed that priming activity of the lip motor area with TMS facilitated the recognition of labials (e.g., /p/) masked by white noise, while stimulating the tongue motor area enhanced the recognition of dentals (e.g., /t/). In their follow-up study (D’Ausilio et al., 2012), they in fact reported that such facilitation effects were only observed when syllables were embedded in noise, not when they were intact. Conversely, temporarily disrupting left premotor cortex activity by rTMS altered the discrimination of plosives in noisy CV syllables (/pa/, /ta/, /ka/; Meister et al., 2007; see also Murakami et al., 2015, and Sato et al., 2009, for similar findings).

Converging evidence of specific motor activity during degraded speech processing comes from Callan and coworkers (2010) who highlighted the contribution of the ventral premotor cortex to distinguish between correct and incorrect phonemes in noisy conditions. Similarly, using a continuum ranging from white noise to CV syllables, Osnes and collaborators (2011) found an increase of fMRI hemodynamic activity in superior temporal regions, with a left-hemisphere bias, as sounds became progressively more recognized as speech. Crucially, the left premotor cortex was specifically activated when sounds became identifiable as speech but were still noisy, whereas temporal cortical activity ceased to increase in this condition. Effective connectivity analyses in the left hemisphere additionally showed bidirectional connections between the premotor cortex and superior temporal sulcus, together with unidirectional transfer from the planum temporale to the premotor cortex (see also Alho et al., 2014). These results confirm that premotor regions selectively come into play when processing degraded though still identifiable speech sounds.

Du and colleagues (2014) reached similar conclusions using MVPA to examine the encoding of syllables that varied in articulatory features and were presented under different perceptual conditions. They showed that while activity in temporal regions exhibited good phoneme categorization only in the absence of noise, activity within premotor regions appeared more robust to distinguish degraded phonemes. These findings therefore suggest that the motor system can help speech processing, in moderately noisy conditions at least. They also align with the view that (pre)motor regions are part of a sensorimotor circuit that simulates articulatory gestures to anticipate sensory outcomes through the use of internal models (see Callan et al., 2004; Grisoni & Pulvermüller, 2022; Rauschecker & Scott, 2009; Skipper et al., 2007; Wilson et al., 2004; but see Hickok, 2012). Such top-down predictive coding likely enhances speech perception when the perceptual condition is challenging, underlining the role of the motor system in compensatory perceptual mechanisms that constrain and facilitate speech comprehension (Callan et al., 2004).

Motor activity has also been reported for other types of speech degradation, from noise-vocoding (Hervais-Adelman et al., 2012) to speech rate acceleration (Adank & Devlin, 2010; Hincapié Casas et al., 2021) and inter-talker variability (Bartoli et al., 2015). In a series of TMS studies, Nuttall and collaborators (2016, 2017) observed that this motor activity occurs irrespective of the nature of the degradation, either extrinsic or intrinsic to the speech signal. TMS applied over the lip motor region induced significantly larger MEPs during the perception of distorted than intact speech sounds, both when the distortion was caused by white noise masking and when it resulted from obstruction of the speaker’s lip and tongue movements. Nuttall et al. furthermore observed larger lip motor activity for labials (/aba/, /apa/) than for dentals (/ada/, /ata/) but only in the distorted conditions (in line with D’Ausilio et al., 2012). Two other findings were of primary interest. First, participants who were better at identifying the degraded syllables showed larger lip MEPs during passive perception, compared to low performers (Nuttall et al., 2016). In other words, stronger motor activity to heard speech was associated with better recognition of degraded speech (see D’Ausilio et al., 2014, for converging results). Second, participants’ hearing sensitivity influenced motor recruitment during perception (Nuttall et al., 2017). Whereas speech motor facilitation was found for noisy speech sounds in participants with better auditory acuity, participants with normal but lower hearing performance showed stronger MEPs for clear speech (see also Du et al., 2016, for similar evidence in younger and older listeners). This suggests that the motor cortex may compensate for impoverished auditory information, resulting either from the signal itself or from a decrease in hearing abilities.

## MOTOR RESONANCE TO FOREIGN PHONEMES

Speech motor areas are recruited for the perception of phonemes in the native language, especially under degraded conditions as discussed in the previous sections. In parallel, the embodiment of phonemes that are not part of the listener’s phonological repertoire has been investigated, although to a much lesser extent (Figure 1). Wilson and Iacoboni (2006) examined the fMRI neural responses in auditory and motor cortices for 25 non-native phonemes (e.g., stops, fricatives, clicks, trills, and nasals) belonging to different languages and varying in producibility for English native speakers. Compared to native phonemes, non-native sounds yielded an increased activity in bilateral superior temporal regions. Interestingly, the more difficult the phonemes were judged to produce, the more the temporal cortices were activated. With regard to the motor cortex, a region-of-interest analysis revealed that, for both hemispheres alike, the ventral premotor cortex was more activated for non-native compared to native phonemes (note that whole-brain analyses revealed (pre)motor activity for all speech sounds vs. rest). In addition, the premotor cortex was found to be functionally connected with superior temporal regions that distinguished non-native from native sounds and that coded for producibility. Wilson and Iacoboni (2006) interpreted their findings in light of internal models instantiated within motor regions to predict the acoustic consequences of the perceived phonemes (see also Callan et al., 2004). Whereas a match between such predictions and the actual sounds would be rapidly obtained for the native language, the repeated and unsuccessful attempts to simulate unknown, non-native speech sounds would account for the greater motor activity observed.

Increased motor cortical activity for non-native phonemes has been corroborated by Schmitz and coworkers (2019) using TMS. The authors probed the lip representation excitability in the left primary motor cortex while Italian participants passively listened to native and non-native German vowels. Echoing Wilson and Iacoboni’s (2006) results in the temporal cortices, they reported a negative correlation between nativeness ratings and the lip motor potentials evoked for vowels: the less the vowel appeared as pertaining to the native repertoire, the higher the excitability in the lip motor representation. The authors suggested a compensatory role of the motor cortex when listening to speech sounds that lack a defined acoustic-motor representation. Such an interpretation fits with the above-reviewed findings on degraded native speech perception (D’Ausilio et al., 2012; Nuttall et al., 2016, 2017) as well as with fMRI studies showing strong bilateral (pre)motor cortical activity for the perception of difficult contrasts in non-native languages (Callan et al., 2003, 2004, 2014). Identification of words starting with the English phonemes /ɹ/ or /l/, which are hardly distinguished by Japanese speakers, even with English experience, has indeed been shown to enhance activity in a bilateral network encompassing articulatory cortical regions in those participants (Callan et al., 2003). Interestingly, when native speakers of English performed the task on these same English phonemes but produced by Japanese speakers, therefore with a foreign accent, strong bilateral premotor involvement was also found (Callan et al., 2014). Converging findings were reported from phonetic training studies. A bilateral network including the inferior frontal gyrus, involved in articulatory processes, was activated after English native adults were trained for 5 hours to identify the Hindi dental retroflex consonant /ɖ/ (Golestani & Zatorre, 2004). This network was highly comparable to that recruited for the processing of the native consonants /d/ and /t/. On the other hand, Callan and colleagues (2004) showed an extension of activity in the premotor cortex, Broca’s area, and supramarginal gyrus (among other cortical and subcortical regions) from the left hemisphere to both hemispheres, after Japanese native speakers were extensively exposed for 1 month to the English difficult /ɹ/-/l/ contrast. Contrary to Golestani and Zatorre’s findings, however, this pattern of activation spread far beyond that observed for the perception of an easy contrast (/b/-/g/, not trained) that also exists in Japanese. The authors interpreted their findings as reflecting the need to establish auditory–articulatory mappings when acquiring new phonemic categories, thus engaging additional neural resources also in the right hemisphere, to ease non-native speech acquisition and perception. Alternatively, the increased motor activation for non-native speech sounds observed across studies could reflect the recruitment of established native articulatory patterns, thus possibly interfering with foreign language processing and learning. Future neuromodulation studies targeting the motor cortex with TMS or transcranial direct current stimulation (tDCS) in pre- and post-training paradigms may help decipher if and when motor regions facilitate or hinder the establishment of non-native phonemic categories.

Altogether, previous findings support the idea that listeners recruit brain regions involved in speech production to process heard speech, especially under adverse auditory conditions (Figure 1). In this view, although not being strictly essential for speech perception, the motor system seems to play a crucial role in speech sensorimotor integration by constraining phonemic categorization, ultimately facilitating speech perception (Callan et al., 2004, 2014; Iacoboni, 2008; Rauschecker & Scott, 2009; Schwartz et al., 2012; Skipper et al., 2007, 2017). Such a functional contribution raises the question of whether processing and learning non-native phonemes could benefit from sensorimotor training. Before presenting recent advances along this line, we will first review the learning paradigms classically developed to support the acquisition of phonemes in a foreign language.

## CLASSICAL LEARNING PARADIGMS FOR FOREIGN PHONEMES: HIGH VARIABILITY PHONETIC TRAINING

Learning speech sounds that are not part of our phonological inventory is challenging, especially in adulthood. Since adult learners cannot rely on robust auditory or articulatory patterns for these newly acquired sounds, they often find them problematic to distinguish from native phonemes. On the production side, this is typically reflected by a non-native way of pronouncing foreign phonemes, a phenomenon commonly experienced as a foreign accent. The proximity between the phonological systems in the native and the foreign languages has been advocated as a major factor that influences learning new language’s phonemes. According to Flege’s speech learning model (Flege, 1995), foreign speech sounds perceived as close to native phonemes tend to be assimilated to their native counterparts, and are therefore less well recognized and produced than more distant foreign phonemes. In other words, the greater the perceptual distance between a non-native speech sound and a native phoneme, the more likely and easily it will form a new phonemic category (see also the perceptual assimilation model; Best, 1994; Best et al., 2001).

Despite these difficulties, learning new phonemes has been shown to benefit from laboratory training based on perception and/or production. In this respect, one of the most common training paradigms used to improve foreign speech sound processing is high variability phonetic training (HVPT; Logan et al., 1991), which is embedded in a pre-test/post-test design. HVPT consists in presenting multiple natural tokens of the target phonemes produced by several native speakers in a variety of phonological environments (e.g., varying adjacent phonemes and/or syllabic positions). Tokens are typically presented from minimal pairs contrasting the native and non-native phonemes, and participants are required to perform a two-alternative forced-choice (2-AFC) identification task with immediate feedback on their response. Exposing learners to a wide range of acoustic–phonetic cues across different phonological environments during training is thought to enhance perceptual learning and thus to promote the development of new phonemic categories. In addition, providing feedback allows to focus the participants’ attention on the crucial cues of the speech sounds under consideration (Logan et al., 1991; but see Vlahou et al., 2012, for more robust learning after implicit training without external feedback). Pre- and post-training performance is assessed with the same identification task but without any feedback. To assess the generalization of learning, both trained and new tokens, produced by the same or by different speakers, are usually included.

Numerous studies have shown improvement of learners’ perceptual performance after 3–4 weeks of HVPT (15–45 training sessions), mostly regarding the English /ɹ/-/l/ contrast that Japanese native speakers struggle to discriminate. The benefits of HVPT furthermore generalized to new exemplars and speakers (Bradlow et al., 1997; Callan et al., 2003; Iverson et al., 2005; Lively et al., 1993, 1994; Logan et al., 1991; McClelland et al., 2002; Shinohara & Iverson, 2018), with (moderate) long-term effects up to 6 months after training (Bradlow et al., 1999; Lively et al., 1994). HVPT can also enhance perceptual performance for other phonological contrasts, such as places of articulation in consonants (Cebrian & Carlet, 2014; Golestani & Zatorre, 2004, 2009; Pruitt et al., 2006), as well as for vowels (Iverson et al., 2012; Nishi & Kewley-Port, 2007, 2008) and tones (Wang et al., 1999, 2003). For instance, in a classical HVPT paradigm varying consonantal contexts and speakers (Lambacher et al., 2005), Japanese native speakers exhibited higher identification of vowels from American English at post-test, in particular for those that were more distant from their native repertoire (/ɔ/ and /ɝ/). Interestingly, perceptual identification training has also proved successful on speech production, despite no explicit articulatory instruction being provided to the learners (see Sakai & Moorman, 2018, for a review). Bradlow and colleagues (1997) reported that the production of words containing /ɹ/ or /l/ by Japanese trainees was rated higher and was better identified by English native speakers after perceptual training than before. In agreement with this study, Lambacher et al. (2005) also showed that, at post-test, the American English vowels produced by Japanese learners were better identified by native speakers, and their spectral overlap was reduced, compared to pre-test. This was especially true for more distant vowels (/ɔ/, /ɝ/ and /æ/) whereas vowels (/ɑ/ and /ʌ/) phonetically similar to their Japanese counterpart (/a/) still showed a large degree of overlap after training. These findings support models of second language acquisition (Best, 1994; Flege, 1995) by revealing greater improvements, both in perception and production, for non-native vowels that share less phonetic features with the native phonological inventory. In addition, they show that transfer of knowledge can occur from perceptual learning to production of non-native phonetic contrasts, highlighting the existence of common auditory-articulatory representations for speech perception and production.

Although HVPT has repeatedly been shown to improve foreign speech sound learning, its effects can vary depending on learners’ native repertoire (e.g., better learning for larger L1 vowel inventory; Iverson & Evans, 2007, 2009), as well as on their perceptual abilities (e.g., detrimental effects for learners with low initial skills; Perrachione et al., 2011; Sadakata & McQueen, 2014). The source of variability required for efficient learning has also been questioned, especially regarding the use of multiple versus single talkers in HVPT. Whereas the meta-analysis by Zhang, Cheng, and Zhang (2021) found a robust advantage of multi-talker over single-talker training, Brekelmans and colleagues (2022) showed in their review that trainees exposed to high variability in voices did not always outperform those exposed to low variability input. In an attempt to carefully replicate the studies by Logan et al. (1991) and Lively et al. (1993) on the English /ɹ/-/l/ contrast, they found a gain in post-test performance, with generalization to new speakers, both for high variability (including five English native speakers) and low variability (with only one speaker) training, considering learners’ initial abilities (see also Xie et al., 2021, for lack of replication of Bradlow & Bent, 2008, on foreign-accented speech). Altogether, it appears that high variability during phonetic training is beneficial for learning and generalization but that this variability does not necessarily need to originate from various speakers as long as multiple tokens of the target phonemes are provided.

In this regard, studies showed that increasing the acoustic variability of temporal or spectral cues that are irrelevant to non-native speech sounds can also boost learning (Iverson et al., 2005; Ylinen et al., 2010; Zhang et al., 2009). Chinese native adults, for instance, learned the English vowel /i/-/ɪ/ contrast better in a modified HPVT design, where acoustic stimuli were temporally exaggerated compared to a canonical HVPT paradigm, despite this temporal manipulation not being informative to distinguish the vowel categories (Cheng et al., 2019; see also Zhang, Cheng, et al., 2021, for a follow-up study). The authors suggested that adding the irrelevant durational cue during training reallocated learners’ attention to the relevant spectral categorical information, which was better extracted, thus improving learning. Phonetic training in noise (e.g., speech-shaped noise, multitalker babble) also proved to benefit foreign phoneme identification (Cooke & Garcia Lecumberri, 2018; Leong et al., 2018). In the HVPT study by Mi and colleagues (2021), Chinese native speakers who learned English vowels embedded in a multitalker babble or presented in quiet (i.e., without noise) outperformed a control group who did not benefit from any training. However, only the group trained with the babble maintained their level of performance 3 months after training. Hence, adding background noise during training can help develop more robust speech representations in the non-native language. According to Mi and colleagues (2021), this may be explained by enhanced top-down attentional processes and/or increased weight of important acoustic cues (in line with Cheng et al., 2019 and Zhang, Cheng, & Zhang, 2021). Given the functional role of motor regions in challenging speech perception, it is also possible, although this was not discussed by the authors, that training in noise may encourage the reliance on motor forward internal models that would benefit non-native phoneme categorization. Additional work is needed to further assess this issue, both on foreign speech sound perception and production (see Mora et al., 2022, for a study on production with HVPT in noise).

## AUDIOVISUAL TRAINING PARADIGMS FOR LEARNING FOREIGN PHONEMES

The above-reviewed HVPT studies focused on purely auditory training, leaving aside visual articulatory information available from lip-reading that otherwise plays an important role in face-to-face communication (Dohen et al., 2010; Hardison & Pennington, 2021; McGurk & MacDonald, 1976). A few other studies have compared the effectiveness of audiovisual and auditory training, and most of them showed an advantage of providing additional visual cues on the perception and/or production of newly learned foreign phonemes (Hardison, 2003, 2005; Hazan et al., 2005; Inceoglu, 2016; Li & Somlak, 2019; Navarra & Soto-Faraco, 2007; Wang et al., 2014). Pereira Reyes and Hazan (2021) found comparable improvement in English vowel identification and production by Spanish native speakers following audiovisual and auditory phonetic training. Remarkably, training only with visual cues (without any auditory input) had the same effects, suggesting that merely attending to lip articulatory gestures during training can promote the learning of non-native phonemes.

Other studies, however, have revealed that the efficiency of audiovisual training may depend on factors such as the informational value of the visual cues and the phonemic contrasts to acquire (Hazan et al., 2006; Ortega-Llebaria et al., 2001; Werker et al., 1992). In their HVPT study, Hazan and colleagues (2005) showed that audiovisual training in Japanese learners benefitted phonemic identification more than auditory training for the labial/labiodental /p/-/v/ contrast for which visual information is highly distinctive. This was not the case for the /ɹ/-/l/ alveolar contrast, which is less visually salient (but see Hardison, 2003) and which showed similar perceptual improvement after audiovisual and auditory training. Better pronunciation of this latter contrast was nevertheless observed after audiovisual than after auditory training, suggesting that information on articulatory gestures improves production (Hazan et al., 2005). In this regard, Massaro and Light (2003) did not report any further improvement in Japanese learners of English when trained with a computer-animated talking head illustrating the internal oral cavity and the precise articulatory gestures for the /ɹ/-/l/ contrast, compared to training with a classical frontal view of the (tutor’s) talking head (see Grauwinkel et al., 2007, and Wik & Engwall, 2008, for supporting evidence). Hence, although multisensory training may foster non-native phonological learning, this is not always the case, especially when visual articulatory information is not salient enough. Considering the potential advantage of supplementary visual information and fully exploiting the embodied nature of speech, new training paradigms integrating manual gestures have emerged to overcome the lack of accessibility of relevant articulatory cues for learning non-native phonemes.

## EMBODIED TRAINING PARADIGMS FOR LEARNING FOREIGN PHONEMES

Spontaneous hand gestures usually come along with speech in all languages and cultures, providing complementary meaning to the auditory verbal input (Goldin-Meadow & Alibali, 2013; Iverson & Goldin-Meadow, 1998; Iverson & Thelen, 1999; McNeill, 1992, 2000; Wagner et al., 2014). This intertwining of speech and gestures arises early during native language development (Goldin-Meadow, 2010; Iverson, 2010), and gestures keep on easing language production in healthy adults and in patients with language and communication disorders (Akbıyık et al., 2018; Clough & Duff, 2020; Hogrefe et al., 2013). Gestures have also been shown to enhance vocabulary learning in a foreign language, mostly when they illustrate the semantic content of target words (i.e., iconic gestures; Gullberg, 2006; Kelly & Lee, 2012; Macedonia, 2014; Macedonia & Klimesch, 2014; see Kühne & Gianelli, 2019, for a review). Performing iconic gestures while speaking can furthermore aid listeners’ word comprehension under moderately adverse conditions, for instance, when the acoustic signal is spectrally degraded (Drijvers & Özyürek, 2017; Drijvers et al., 2018), both in young and in older adults (Schubotz et al., 2021). Interestingly, such benefit has also been reported in highly proficient non-native listeners, albeit to a lesser extent than in native listeners (Drijvers & Özyürek, 2018, 2020; Drijvers, Vaitonytė, & Özyürek, 2019; Drijvers, van der Plas, et al., 2019). It was proposed that a more intelligible auditory signal is required for non-native listeners to optimally map this information with the semantic information conveyed by the manual gestures, and thus to benefit from these extra cues. Although these studies examined word-level comprehension in high-proficiency non-natives, and despite the potential boosting effect of noise in learning foreign phonemes (e.g., Cheng et al., 2019; Mi et al., 2021), these findings deserve further attention for training paradigms combining speech with manual gestures to promote non-native speech sound learning. The last decade has indeed seen a growing interest in gestural learning for foreign phonemes; however, mixed results have been reported (e.g., Amand & Touhami, 2016; Baills et al., 2019; Hirata et al., 2014; Li et al., 2020, 2021; Xi et al., 2020; Zheng et al., 2018).

### Pitch

Several studies showed that manual pitch gestures, mimicking the fundamental frequency (F0) contour of speech, can facilitate word learning in non-native tonal languages. Learners who observed and/or imitated upward and downward hand gestures to depict, respectively, high- and low-frequency pitch contours during training indeed improved their perception or pronunciation of lexical tones (Baills et al., 2019; Zhen et al., 2019; Zheng et al., 2018; see also Hannah et al., 2016, 2017, for perception paradigms without any training). Morett and Chang (2015) failed to show any gain in Mandarin tone identification in English native speakers trained by imitating pitch gestures compared to a non-gestural training. A subsequent word-meaning association task, however, revealed better performance in the gestural condition, supporting the advantage of metaphorical pitch gestures in learning foreign words that differ in lexical tones (see also Morett, 2023, for an EEG study). Notably, enacting pitch gestures might not be more beneficial to tone learning than merely observing them, as shown by the few studies that directly compared the two modalities (Baills et al., 2019). Still, at the suprasegmental level, the beneficial effects of arm/hand gestures were shown on the perception (Kelly et al., 2017) and pronunciation (Yuan et al., 2019) of intonational patterns, as well as on the accentedness of foreign speech (Baills & Prieto, 2023; Baills et al., 2018; Gluhareva & Prieto, 2017). In Kushch’s (2018) work, Catalan learners produced Russian words with a better accent, as evaluated by Russian native speakers, after training that involved beat gestures highlighting speech prominence. This was particularly the case if the gestures had been imitated rather than observed. Along the same line, Baills, Santiago, et al. (2022) found that Catalan learners improved in French accent in an oral reading task after training with sentence-level prosodic (pitch) imitated gestures (but see Baills, Alazard-Guiu, & Prieto, 2022, for contradictory findings).

### Vowels

Besides prosodic patterns, embodied training paradigms have also been developed to encode segmental information such as vowel-length contrasts (see Table 1 for an overview of studies on gestural paradigms for foreign vowels). Within this scope, beat and durational gestures have mostly been used to train discrimination between short and long vowels, respectively, but consensual evidence for their benefits is so far lacking. Whereas beat gestures (McNeill, 1992) consist in nonreferential up-and-down movements associated with prosodic prominence, durational gestures are typically represented with horizontal hand-sweep movements. Hirata and Kelly (2010) investigated the effect of lip movements and/or hand gestures in English native adults learning Japanese vowel-length contrasts such as /i/-/iː/. Four types of trainings were proposed: (1) auditory input, (2) auditory input and visual lip movements, (3) auditory input and visual hand gestures, or (4) auditory input and both visual lip and hand gestures. In the two hand-gestural conditions, the instructor produced short and long vowels concurrently with, respectively, a hand flick (beat gesture) and a prolonged horizontal hand sweep (durational gesture) that the participants had to observe. Results revealed better vowel identification in all training groups, but with larger improvement after the audio-lip training. Hence, providing hand gestures during training did not particularly help learners in perceiving the length difference between vowels (see also Kelly et al., 2017, and Kelly & Hirata, 2017, for similar conclusions). One possible interpretation for these findings is that mixing the two types of gestures during training may have prompted learners to focus more on gesture discrimination than on the auditory speech input (but see Hirata et al., 2014, who found similar results with an additional training condition based on the rhythmicity of Japanese moras using hand flicks only, one for short vowels and two for long vowels). The potential lack of obvious correspondence between the hand flick gesture and the short vowel for English native listeners, as well as the possibility that beat gestures may benefit suprasegmental processing in the native language but not non-native segmental processing (Hubbard et al., 2009; Krahmer & Swerts, 2007), may also account for the poor efficiency of the hand gestures in these two studies. Li and colleagues (2020) on the other hand reported that imitating horizontal hand-sweep gestures whose duration mimicked vowel length during training improved the distinction of Japanese short and long vowels (/e/-/eː/ and /o/-/oː/) in Catalan adults. This advantage of durational gestures over training without gestures was, however, found only for non-native phoneme production (see also Li et al., 2023, for effects of prosodic gestures on the production of French front-rounded vowels by Catalan speakers). Identification performance on the contrary improved similarly following the two types of training. Hence, in line with the previously mentioned work (Hirata & Kelly, 2010; Hirata et al., 2014), hand gestures failed to improve the perception of vowel-length contrasts, whether these gestures were merely observed or reproduced. This may be explained by the major challenge in acquiring this type of contrast compared to pitch contrasts for non-native speakers (Hirata, 2015). Despite the encouraging results on the production side at least, and the fact that durational gestures are spontaneously used to teach foreign language pronunciation in classrooms (Smotrova, 2017, for a review), further empirical evidence is needed to target the right gestures and fully support the beneficial role of hand gestures on the learning of non-native durational vowel contrasts.

**Table 1.**
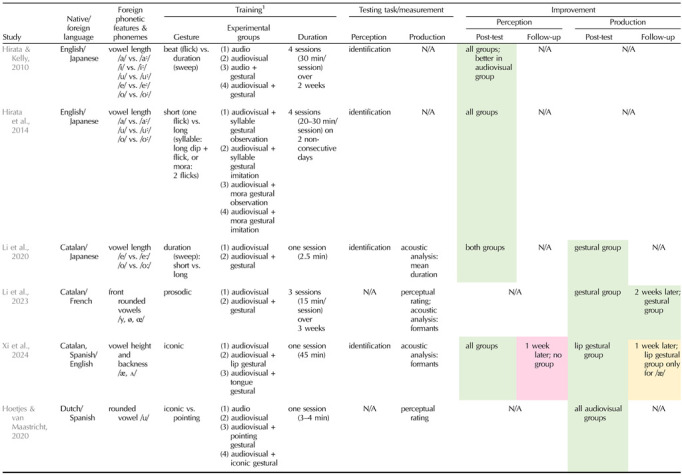
Overview of studies using gestural learning paradigms for foreign vowels

^1^ Learners were required to repeat phonemes during training only in Li et al. (2020, 2023) studies.

Green cells indicate that gestural training improved learning of foreign speech sounds, whereas yellow and red cells stand for, respectively, limited and lack of improvement. N/A = not tested in the study; min = minute(s).

### Phonetic Features of Consonants

What about phonetic features? Can manual gestures that explicitly code for place or manner of articulation help with learning non-native speech sounds? A handful of recent studies have tackled this issue, with generally promising results, in particular on speech sound production (e.g., Amand & Touhami, 2016, for unreleased plosives; Ozakin et al., 2023, for fricatives; Xi et al., 2023, for vowel lip aperture; see Table 2 for an overview of studies targeting consonants with gestural learning paradigms). Xi and colleagues (2020) trained Catalan adults to learn Chinese plosive and affricate consonants contrasting on aspiration either while observing a fist-to-open hand gesture illustrating the extra air burst for aspiration or without any manual gesture. Notably, the fist-to-open hand gesture closely mimicked the production (and perception) of the aspirated plosives (sudden opening of the fingers illustrating the quick opening of the lips and prominent air burst), whereas it less well matched the aspirated affricates characterized by a more gradual and less prominent air release. After a 5-minute training session without any feedback, results revealed better pronunciation of the aspirated plosives only in the gesture group. No gestural advantage was found for the aspirated affricates. In contrast, identification performance for both plosives and affricates did not benefit from hand gestures compared to the no-gesture training condition (in line with previous work on vowels; Hirata & Kelly, 2010; Hirata et al., 2014; Li et al., 2020). These findings emphasize that only manual gestures that appropriately reflect the phonetic features of non-native phonemes may foster their acquisition in adults and improve their pronunciation. A follow-up study (Li et al., 2021) confirmed the importance not only of the addition of manual gestures during training but also of the accuracy of the learners’ gestural performance. Catalan adults who appropriately imitated bimanual fist-to-open hand gestures while repeating Mandarin aspirated plosives during training indeed improved more on uttering these phonemes than learners who poorly imitated the same hand gestures. This was reflected by enhanced voice onset time (VOT) values and better rating of the trainees’ pronunciation by Mandarin native speakers in the well-performed gesture group at post-test (immediately following the one-session training) as well as 3 days later. By contrast, in the poorly performed gesture group, VOT did not change at post-test and the benefit of hand gestures on the rated pronunciation was no longer seen at the delayed post-test. The quality of the imitated gestures is therefore crucial to yield positive effects of embodied training on learning and maintaining non-native phoneme production, pointing to the need of assessing learners’ gestural performance as well as of designing paradigms with adequate gestures.

**Table 2.**
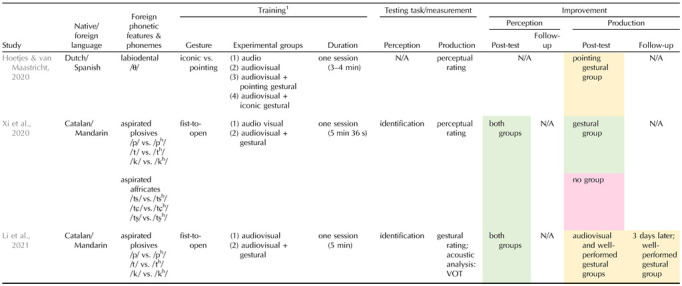
Overview of studies using gestural learning paradigms for foreign consonants

^1^ Learners were required to repeat phonemes during training only in Li et al. (2021).

Green cells indicate that gestural training improved learning of foreign speech sounds, whereas yellow and red cells stand for, respectively, limited and lack of improvement. N/A = not tested in the study; min = minute(s); s = second(s); VOT = voice onset time.

The complexity of the manual gestures and the fact that they stand for visible or nonvisible articulatory features also seem to impact learning efficiency. In the study by Hoetjes and van Maastricht (2020), Spanish adults learned to produce two Dutch phonemes that are part (/u/) or not (/θ/) of their native repertoire and that require new phoneme–grapheme correspondences to be acquired. The easy vowel /u/ was better produced after training based on the observation of an iconic hand gesture illustrating the rounding of the lips rather than on the observation of a simple pointing gesture to the mouth. The reverse was found for the more challenging consonant /θ/: learning was hindered by an iconic gesture indicating to push the tongue between the teeth, while it was more efficient with the (simpler) pointing gesture. The authors suggested that manual gestures reflecting phonetic features may help phonemic learning only when processing demands are not too high, such as for the easy vowel /u/. When processing cost increases, for example, to acquire a non-native phoneme outside the native phonological inventory, providing complex hand gestures may be detrimental to learning (see Kelly & Lee, 2012, for similar arguments). Notably, even though Hoetjes and van Maastricht (2020) did not discuss this point, the fact that the gestures illustrated the lips or the tongue, namely, articulators that are directly visible or not for the learners, may also have affected the effectiveness of learning.

As a matter of fact, Xi and coworkers (2024) revealed that observing lip-related gestures during training facilitated the production of the English vowels /æ/ and /ʌ/ by Catalan-Spanish adults more than observing gestures mimicking tongue shape within the mouth. These two vowels differ in both the degree of lip aperture and tongue position along the anteroposterior plane, and they tend to be assimilated to /a/ by Spanish speakers. Gestural training therefore involved either a one-handed gesture depicting the lip aperture needed to produce the vowels, or a bimanual gesture representing tongue backness relative to a reference point (as well as lip aperture from the distance between the two hands). A control group was trained in a classical audiovisual condition without hand gestures. Identification improved comparably in all training groups, in line with the limited effects of gestural paradigms on perception (e.g., Hirata et al., 2014; Li et al., 2020, 2021; Xi et al., 2020). For production, however, results revealed that the lip-related gesture helped the learners to adjust their lip aperture (as measured by formant values) for non-native vowels more than the tongue-related gesture and nongestural conditions. The efficacy of the training to adjust tongue position was, on the contrary, limited and similar across the three groups. Hence, hand gestures that encode visible articulatory features, such as lip aperture, may be more beneficial than gestures coding for nonvisible features, involving the tongue in particular, as the latter may potentially increase the processing demands. Indeed, manual gestures mimicking the tongue shape do not match visual facial information and may therefore create some kind of incongruency for the learners as opposed to lip-related gestures that give complementary congruent information about the way phonemes are produced. Notably, as pointed out by Xi and colleagues (2024), the lack of feedback in the training paradigm in their study, as well as in the work by Hoetjes and van Maastricht (2020), might also explain the limited learning advantage of tongue-related hand gestures. In fact, two studies, in a classroom (Lan & Wu, 2013) and in a clinical setting (Rusiewicz & Rivera, 2017), reported better non-native or native consonant pronunciation after participants imitated hand gestures that illustrated the shape of the tongue. The fact that learners only observed the manual gestures in Xi et al. (2024) and Hoetjes and van Maastricht (2020) suggests that actually performing the gestures may be a key ingredient for efficient learning. Although this interpretation is in line with most studies on vocabulary learning or more general cognitive skills (e.g., Goldin-Meadow et al., 2009; Macedonia et al., 2011; but see Kelly et al., 2014), the few studies that compared observation and imitation for foreign phoneme learning have provided mixed results (advantage of imitation over observation: e.g., Baills et al., 2019; Kushch, 2018; no advantage: e.g., Hirata et al., 2014; Kelly et al., 2014). While manual gestures illustrating nonvisible articulatory features may be difficult to integrate with incongruent visual facial cues, they might still be effective when actively and correctly imitated, highlighting the importance of embodied practices for enhancing phonetic acquisition.

Overall, current training paradigms offer a limited framework that varies in effectiveness with regards to the various gestures employed and the few phonemes investigated across different languages. Yet, there are some implications for future training paradigms to build new phonemic categories so as to improve both perception and production in a non-native language. Manual gestures that emphasize distinctions between phonemes should precisely represent the articulatory features of the target foreign speech sounds to acquire. Some challenges still remain though, for instance, for those articulatory features that are not directly visible (e.g., tongue position or shape) and that did not benefit so well from manual gestures during training, at least when these were merely observed, or on the perception side. Assessing the motor performance of learners for these gestures during the training phase could also be crucial to maximize learning efficacy. In addition, longer training paradigms, currently absent in the literature to the best of our knowledge, may be beneficial in strengthening the link between manual gestures and perceived articulatory features, thereby further improving the perception and production of foreign phonemes.

## CONCLUSION

We provided an overview of the literature on how the motor system contributes to both native and non-native speech perception, as well as how learning non-native speech sounds can benefit from embodied multisensory information. Current neuroimaging evidence indicates that the (pre)motor regions underlying speech production are also engaged in speech perception, underscoring the sensorimotor foundation of speech. Somatotopic motor cortical activity linked to distinct articulatory features further supports the embodied nature of phoneme perception. This motor resonance occurs particularly under challenging perceptual conditions when auditory information is degraded, as well as in the context of non-native speech. Given the motor system’s involvement in decoding articulatory features of perceived phonemes, the potential benefits of sensorimotor-based training for learning become evident. Whereas training paradigms that introduce high phonemic variability can enhance perceptual and production skills in foreign languages, multisensory learning protocols, such as training with manual gestures, appear as a promising venue to bolster the acquisition of non-native speech sounds. The limited number of studies, together with the effects restricted to production or to certain phonetic features, however, warrant further scrutiny to fully attest the benefits of gestural learning. Future research should implement longitudinal paradigms to assess whether both the perception and production of foreign speech sounds benefit from gestural training. In addition, the lack of neuroimaging studies on this topic leaves a critical gap in understanding how gestural training may fine-tune articulatory representations of non-native speech sounds within the motor cortex. Studies using TMS or tDCS to stimulate motor cortex excitability may also provide further evidence to decide on the causal role of these brain regions in non-native language acquisition and processing. This, in turn, will allow the refinement of training protocols to optimize the learning process as well as provide valuable insights into the role of embodied experiences during learning and development.

## FUNDING INFORMATION

Tzuyi Tseng, LabEx ASLAN (https://dx.doi.org/10.13039/501100011602), Award ID: ANR-10-LABX-0081; ANR-11-IDEX-0007. Jennifer Krzonowski, LabEx ASLAN (https://dx.doi.org/10.13039/501100011602), Award ID: ANR-10-LABX-0081; ANR-11-IDEX-0007. Claudio Brozzoli, Agence Nationale de la Recherche (https://dx.doi.org/10.13039/501100001665), Award ID: ANR-19-CE28-0015. Claudio Brozzoli, Appel à Projets Pluridisciplinaires Interne (APPI), Université Lumière Lyon 2. Claudio Brozzoli, James S. McDonnell Foundation (https://dx.doi.org/10.13039/100000913), Award ID: doi.org/10.37717/2021-3101. Alice C. Roy, Agence Nationale de la Recherche (https://dx.doi.org/10.13039/501100001665), Award ID: ANR-19-CE28-0015. Alice C. Roy, Appel à Projets Pluridisciplinaires Interne (APPI), Université Lumière Lyon 2. Alice C. Roy, LabEx ASLAN (https://dx.doi.org/10.13039/501100011602), Award ID: ANR-10-LABX-0081; ANR-11-IDEX-0007. Véronique Boulenger, Agence Nationale de la Recherche (https://dx.doi.org/10.13039/501100001665), Award ID: ANR-19-CE28-0015. Véronique Boulenger, Appel à Projets Pluridisciplinaires Interne (APPI), Université Lumière Lyon 2. Véronique Boulenger, LabEx ASLAN (https://dx.doi.org/10.13039/501100011602), Award ID: ANR-10-LABX-0081; ANR-11-IDEX-0007.

## AUTHOR CONTRIBUTIONS

**Tzuyi Tseng**: Conceptualization; Visualization; Writing – original draft; Writing – review & editing. **Jennifer Krzonowski**: Conceptualization. **Claudio Brozzoli**: Conceptualization; Funding acquisition; Writing – review & editing. **Alice C. Roy**: Conceptualization; Funding acquisition; Project administration; Writing – review & editing. **Véronique Boulenger**: Conceptualization; Funding acquisition; Project administration; Supervision; Writing – original draft; Writing – review & editing.

## DATA AND CODE AVAILABILITY STATEMENTS

This study did not generate any new data or code.
